# Kinetic Resolution of Racemic Mixtures via Enantioselective Photocatalysis

**DOI:** 10.1021/acsami.1c12216

**Published:** 2021-08-11

**Authors:** Nitai Arbell, Kesem Bauer, Yaron Paz

**Affiliations:** † The Russell Berrie Nanotechnology Institute, Technion-Israel Institute of Technology, Haifa 3200003, Israel; ‡ The Wolfson Department of Chemical Engineering, Technion-Israel Institute of Technology, Haifa 3200003, Israel

**Keywords:** chiral resolution, enantioselectivity, photocatalysis, molecular imprinting, modified photocatalysts, molecular recognition, atomic layer deposition, ALD

## Abstract

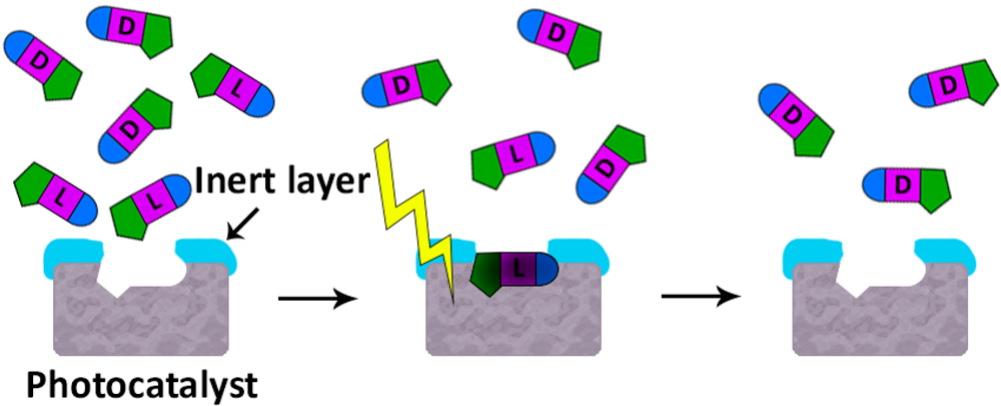

Despite the increasing demand for enantiopure drugs in the pharmaceutical industry, currently available chiral separation technologies are still lagging behind, whether due to throughput or to operability considerations. This paper presents a new kinetic resolution method, based on the specific adsorption of a target enantiomer onto a molecularly imprinted surface of a photocatalyst and its subsequent degradation through a photocatalytic mechanism. The current model system is composed of an active TiO_2_ layer, on which the target enantiomer is adsorbed. A photocatalytic suppression layer of Al_2_O_3_ is then grown around the adsorbed target molecules by atomic layer deposition. Following the removal of the templating molecules, molecularly imprinted cavities that correspond to the adsorbed species are formed. The stereospecific nature of these pores encourages enantioselective degradation of the undesired species through its enhanced adsorption on the photocatalyst surface, while dampening nonselective photocatalytic activity around the imprinted sites. The method, demonstrated with the dipeptide leucylglycine as a model system, revealed a selectivity factor of up to 7 and an enrichment of a single enantiomer to 85% from an initially racemic mixture. The wide range of parameters that can be optimized (photocatalyst, concentration of imprinted sites, type of passivating layer, etc.) points to the great potential of this method for obtaining enantiomerically pure compounds, beginning from racemic mixtures.

## Introduction

Chirality is an unusual aspect of chemistry, a seemingly minor detail with surprisingly far-reaching consequences in physical, chemical, and biological systems.^1,2^ Despite their great similarity, oppositely handed enantiomers of pharmaceutical compounds are known to exhibit wildly different interactions with biological systems, which are themselves inherently chiral. Proteins and peptides are all chiral structures, encoded in chiral DNA strands that are responsible for their biosynthesis.^2,3^ This gives rise to highly spatially selective interactions between these constructs and molecular substrates, as notoriously demonstrated by the thalidomide catastrophe. This drug, prescribed to pregnant women throughout the 1950s, exhibits strong antiemetic and sedative properties for its + enantiomer, while its – enantiomer has significant teratogenic effects, which culminated in tens of thousands of children being born with birth defects due to prenatal exposure.^1,4^ Approximately 50% of drugs on the market today contain at least one chiral center. Many of these compounds exhibit pharmacological asymmetry: i.e., they have one pharmaceutically useful enantiomer (known as a eutomer) and one or more enantiomers that are nonbeneficial or even harmful (distomers).^5,6^ Despite the documented pharmacological asymmetry, some of these compounds are still sold as racemates, even as research shows significant benefits of enantiopure formulations.^7^ Chiral compounds are also of importance in the fields of agriculture and optics.^8,9^

The challenge, however, is in obtaining chirally pure compounds, as both enantiomers respond to most chemical and physical processes identically. This is usually accomplished by the separation of racemic or scalemic mixtures of enantiomers or, alternatively, by directly synthesizing the desired enantiomers using asymmetric synthesis methods.^5,7,10^ While they are extremely useful for many applications, both approaches have their limitations. Asymmetric synthesis, although highly efficient in terms of feedstock, requires the tailoring of complex, multistep, stereoselective reactions, can be relatively expensive, and may also require additional purification steps to reach sufficient optical purity.^7,10^ Separation methods, based on the spatial differences between the enantiomers, require extensive development and optimization: namely, selecting an appropriate separation technique for a specific compound and optimizing its parameters, such as elution order and resolution or crystallization phase. As a general rule, the optimized parameters are hard to predict.^11−13^

One way to overcome this lack of predictable response is through chiral molecular imprinting: i.e., the inclusion of an enantiomerically pure template into the separating matrix to achieve chiral recognition. Chiral imprinting in organic polymers was proposed as an alternative approach to the common chiral chromatography columns, as well as to membrane-based and other retention-based separation techniques.^7,14−17^ Chirally imprinted polymers have also been used for sensing applications.^18,19^ Nevertheless, imprinted polymers may lack structural stability, especially at elevated temperatures.^20^ Molecularly imprinted inorganic structures, although typically more difficult to prepare due to harsher synthetic conditions, making retention of the templated structure more challenging, have in fact been developed previously.^21,22^ Imprinted oxides have been shown to induce spatial resolution for adsorptive, catalytic, and electronic applications.^23,24^ A third group of processes, which can be employed either pre- or postsynthesis, is called kinetic resolution. This is a group of many different reactions which selectively favor one enantiomer over the other, for either the production of the target molecule or its further reaction into easily separable products.^25,26^

The use of photocatalysts, especially anatase-phase titanium dioxide, for the degradation of organic compounds is well documented, particularly in environmental sciences and water treatment technologies. It relies on the light-dependent generation of electron–hole pairs that participate in complex redox reactions, usually mediated via radical species.^27−30^ Molecular imprinting in photocatalytic matrices has been shown to induce selectivity for the degradation of the templating species over homologous alternatives, but alas, not for chiral-selectivity applications.^31,32^ It has been shown that coating with ultrathin layers, for example by self-assembly or atomic layer deposition (ALD), may dramatically affect the properties of photocatalysts, by altering the number of carriers arriving at the surface as well as by controlling the adsorption of reactants and the desorption of products.^33,34^ In this context, our work relies on the pioneering work of Canlas et al., who presented a photocatalytic size-exclusion selectivity effect induced by templating *p*-*tert*-butylcalix[4]arene.^35^

In what follows, a kinetic resolution method for enriching a mixture of enantiomers is presented. The method is based on imprinting chirally pure templating species on the surface of a photocatalyst together with mitigating the activity around the templated sites by growing inactive ultrathin layers using ALD. The method is potentially generic in the sense that it is not limited to a specific compound or enantiomer, since the templating molecule may be altered, under some restrictions, at will.

**Figure 1:**
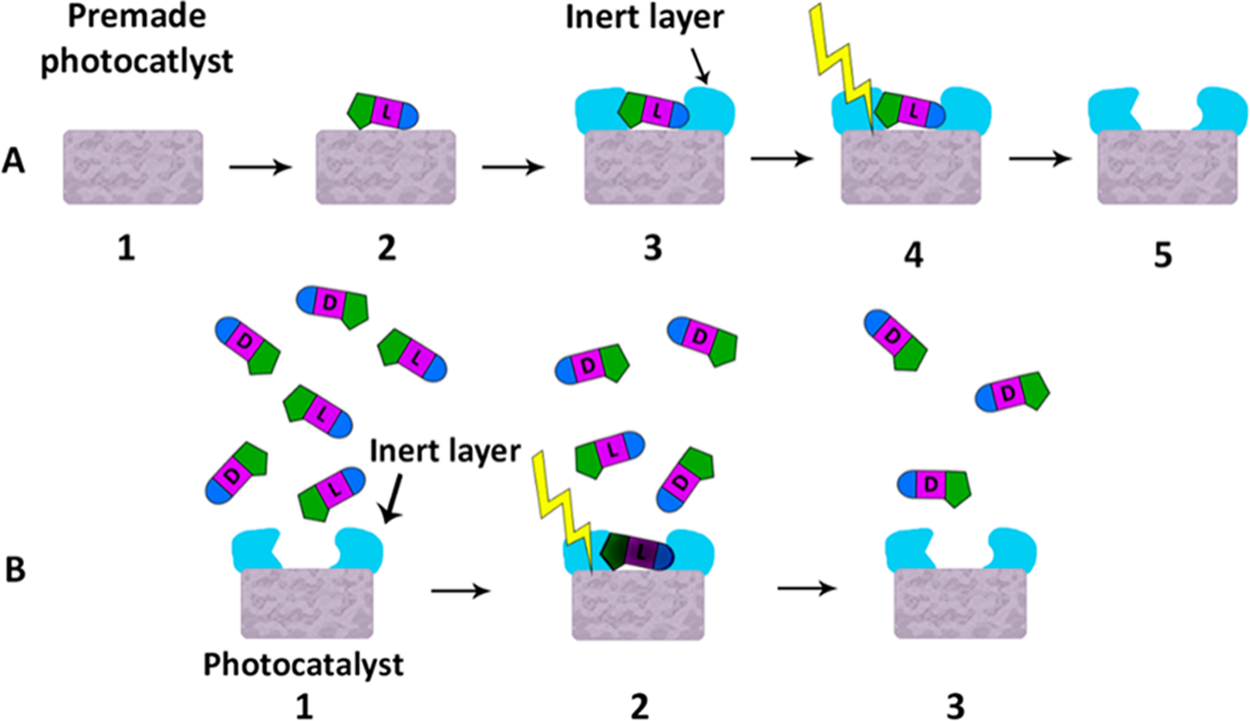
(A) Synthetic scheme for the preparation of a photocatalytic enantioenriching device: (1) photocatalytic matrix; (2) adsorption of a templating enantiomer; (3) growth of an alumina layer by ALD; (4) removal of the templating molecule by UV–ozone cleaning; (5) prepared device. (B) Use of the preprepared PED to obtain enantioenrichment of a racemic mixture.

**Figure 2:**
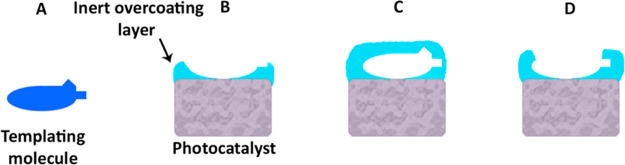
Cartoon describing possible situations during the deposition of an overcoating layer: (A) the templating molecule (the distomer); (B) a PED containing an overcoating layer that is too thin; (C) a PED containing an overcoating layer that is too thick, hence overbridging the cavity; (D) a PED having an optimal overcoating layer.

**Figure 3:**
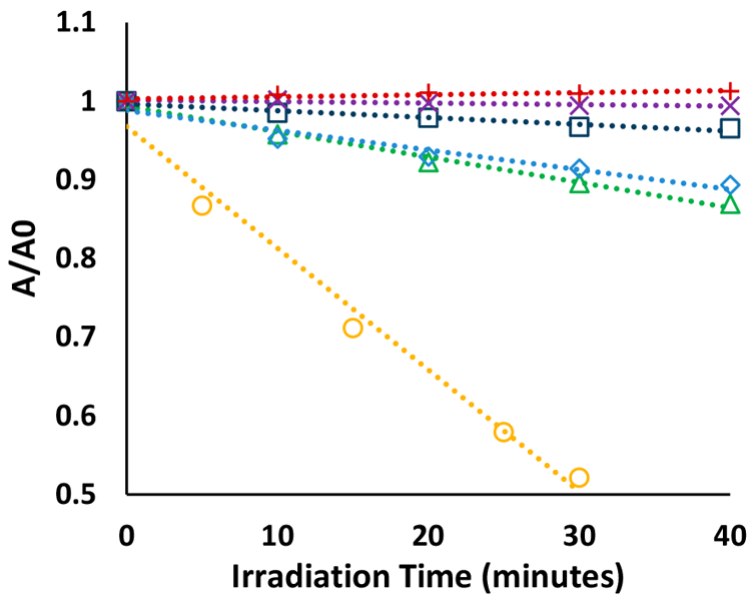
Normalized absobance of a thin layer of stearic acid deposited on the photocatalytic films as a function of UV exposure time for different overcoating cycle numbers and temperatures. Open circles represent no overcoating, triangles, diamonds, squares, and crosses represent overcoating by 4, 6, 8, and 12 cycles at 50 °C, respectively, and X represents overcoating by 8 cycles at 60 °C.

**Figure 4:**
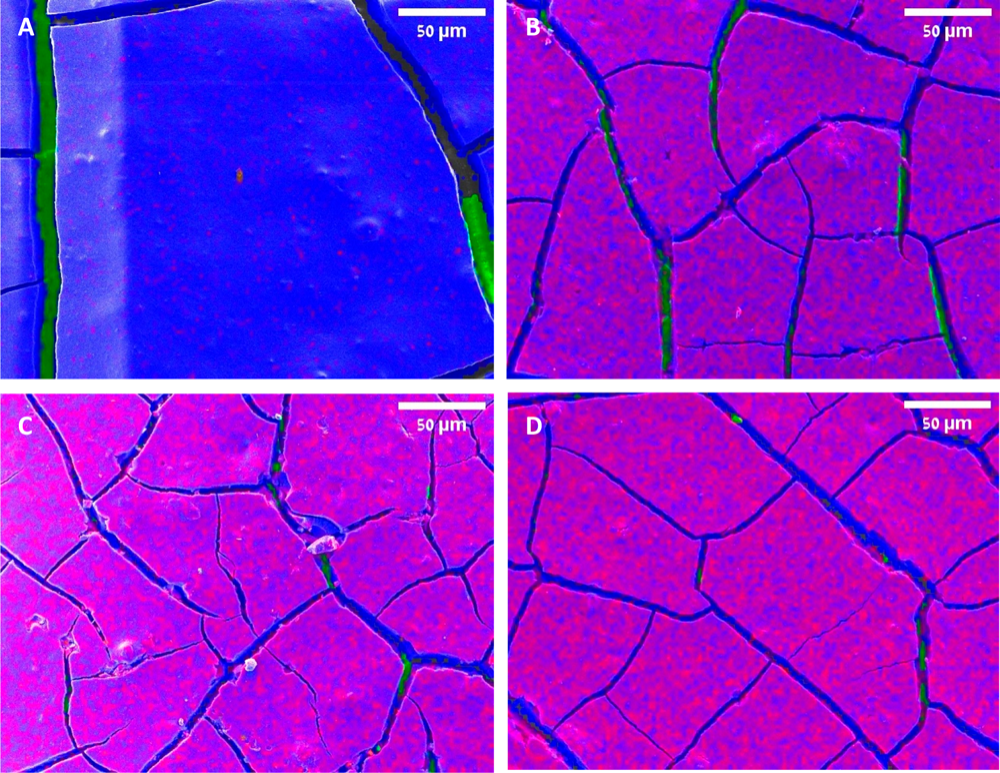
SEM-EDS images of PEDs: (A) TiO_2_ film on glass without any overcoating; (B) overcoated, imprinted plates with 6 ALD cycles; (C) overcoated, imprinted plates with 10 ALD cycles; (D) overcoated, imprinted plates with 14 ALD cycles. Color legend: red, aluminum; blue, titanium, green, silicon from the underlying glass substrate.

**Figure 5:**
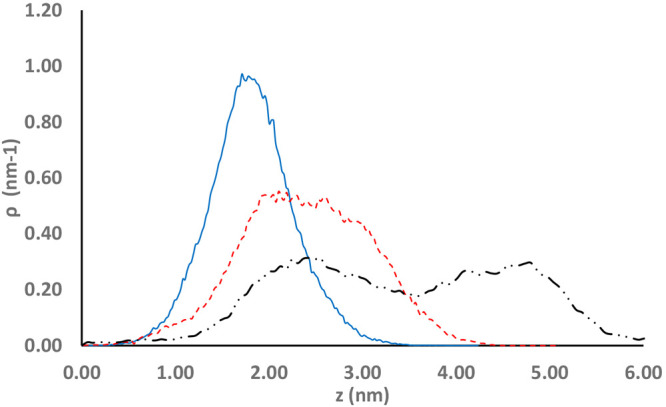
Height distribution functions of the surface of the PEDs as obtained from AFM imaging measured over 50 nm × 50 nm areas. Here, *z* represents the height relative to the minimal height within the frame, while ρ is the prevalence. The solid blue line denotes the coated, nonimprinted sample (+,−), the dashed red line denotes the coated, l-imprinted sample (+,l) templated with an 0.25 mg/mL solution of l-LeuGly, and the dot-dashed black line denotes the l-imprinted sample templated with an 0.5 mg/mL solution of l-LeuGly.

In the specific example demonstrated in this paper, the templating molecule (leucylglycine, LeuGly; Figure S1) is first adsorbed on the surface of the photocatalyst (Figure 1A-1,2). Then, an inert ultrathin layer of aluminum oxide is grown around it (Figure 1A-3). Since in this paper the photocatalyst is in the form of a film coating, the inert ALD layer is hereby termed an “overcoating layer”. Removal of the templating molecule, for example by UV–ozone or plasma (Figure 1A-4), completes (Figure 1A-5) the preparation stage of this photocatalytic enantioenriching device (PED). During operation, the PED is placed in a solution containing a racemic mixture. Preferential degradation of the enantiomers corresponding to the imprinted sites ends in relative enrichment of the solution with the counterenantiomer (Figure 1B).

## Experimental Section

Glass slides, onto which films of TiO_2_ were deposited, served as the starting substrates. This configuration was chosen, despite the low specific surface area, due to its simple preparation procedure. LeuGly, a small dipeptide, was chosen as the model molecule due to its single chiral center, the variety of functional groups it has, allowing for several possible interactions with the matrix, and its commercial availability as an enantiopure compound. Al_2_O_3_ was chosen as the activity-dampening layer due to its significantly larger band gap relative to that of TiO_2_, its favorable band locations, the relatively small lattice mismatch with that of anatase-phase titania, and, above all, its suitability for low-temperature, thermal ALD growth with mild oxidizing reagents such as water.^36,37^

### Activity Suppression

Testing the passivation capabilities of ALD-grown Al_2_O_3_ thin films on TiO_2_ was performed by forming thin layers (∼60 nm) of anatase-phase titania on silica plates using a previously developed spin-coating sol–gel method.^38^ Part of the plates were overcoated with Al_2_O_3_ by thermal ALD, using a MVD100E apparatus (SPTS Ltd.).

The ALD procedure comprised of the following stages: introducing the slides into the reaction chamber, pumping down to less than 1 mTorr, introducing nitrogen to obtain a working pressure of 20 mTorr, introducing 1 Torr of trimethylaluminum (TMA) for 1 s, purging 5 times with nitrogen, pumping down to the working pressure, introducing 1 Torr of H_2_O for 1 s, purging 10 times with nitrogen, and pumping down to the working pressure, thus ending the deposition of a one-cycle overcoating layer. This process was repeated according to the predesigned number of layers.

The default temperature during the process was 50 °C. Various thicknesses were tested, controlled by altering the number of overcoating cycles (0, 4, 6, 8, 12). In addition, an eight-layer sample was grown at 60 °C in order to gain insight with regard to the effect of surface temperature. To test the activity suppression capabilities of the overcoating layer, the degradation kinetics of stearic acid were tested, following a formerly reported procedure,^39^ allowing simple data analysis due to an initial zero-order rate law and easy concentration monitoring using FTIR.^33,40^ Here, a 5 mg/mL stearic acid solution in methanol was deposited and spun twice at 2500 rpm for 2 min. All plates were then individually placed at a distance of 15 cm under a Blakray 100 W, 365 nm UV lamp for 40 min, with measurements taken every 10 min. The kinetics were deduced by monitoring changes in the IR absorption C*H*_2_(*a*) peak at 2916 cm^–1^.

### PED Preparation

PEDs comprised of thick TiO_2_ films on 25 × 12.5 mm glass slides, overcoated with Al_2_O_3_, were prepared by the following procedure. Glass microscope slides (Marienfield) were cleaned by washing in chloroform, ethanol, and deionized water. A mixture of 9.2 g of P25 titania powder (Degussa) and 0.6 g of X-500 titania suspension (TiPE Ltd.) in 12 mL of deionized water was thoroughly (15 min) sonicated (MRC, DC80H) and applied on the glass substrates using the doctor blade method. The deposited thick films (15 μm) were then calcined at 450 °C for 5.5 h to improve adhesion. Next, 300 μL of a 0.25 mg/mL solution of either l-LeuGly (Sigma-Aldrich) or d-LeuGly (Santa Cruz Biotechnology) in deionized water was administered on each of the imprinting-designated slides and spun at 4000 rpm for 80 s. Part of the TiO_2_-coated slides, termed hereby as (−,−), were left aside without LeuGly, All of the LeuGly-containing plates and half of the (−,–-) slides were overcoated with 10 cycles of Al_2_O_3_ ALD, according to the procedure described above. Here, the deposition temperature for all PEDs was 50 °C. Finally, the alumina-overcoated plates were UV–ozone cleaned for 15 min (UVOCS Ltd.) to remove the templating molecules prior to reaction. The prepared PEDs were divided into four groups: nonovercoated nontemplated plates denoted as (−,−), overcoated nontemplated plates denoted as (+,−), overcoated l-templated plates (+,l) and overcoated d-templated plates (+,d). The photocatalytic thick film and overcoating aluminum oxide layers were analyzed using XRD (Rigaku, MiniFlex II), XPS (Thermo VG Scientific, Sigma Probe), and SEM+EDS (Zeiss, Ultra-Plus HRSEM).

Additional ultrahigh-resolution AFM measurements were carried out on PEDs prepared using the same sol–gel procedure used in Activity Suppression. Here, the same templating procedure described above for the P25-containing slides (spin-coating with the templating molecules followed by 10 ALD cycles of Al_2_O_3_) was performed. One additional sample was made with a templating solution containing 0.5 mg/mL of l-LeuGly rather than the 0.25 mg/mL used elsewhere. This type of PED was chosen, as its corrugation is significantly lower than that of the P25-based PEDs. Such low corrugation is a must in order to sense molecular-level cavities. During the AFM measurements of these samples, the plates were first cleaned for 5 min with oxygen plasma and their surface was wetted with a 0.1 M NaCl solution to remove air pockets. This was followed by a quasi-static 4 h stage approach to minimize signal drift, in an in-house-upgraded ultrahigh-resolution AFM setup, reported previously.^41^

### Single-Enantiomer Kinetics

The photocatalytic degradation kinetics of each enantiomer was performed in a reaction vessel comprised of a perforated-bottom 50 mL beaker in which one photocatalytic plate was placed in each experiment. The perforated beaker was introduced into a larger beaker containing a stirring bar, allowing continuous mixing of the solution during the reaction. A glass cover was used to minimize evaporation.

All tests were conducted with 100 mL of an aqueous solution (0.5 mg/mL) of enantiomerically pure (l or d) LeuGly. The photocatalytic plate was placed 15 cm below a Blakray 100 W 365 nm lamp, following adsorption in the dark for 20 h. Each plate was used twice: first with one enantiomer and then with the second. Care was made to alter the sequence of the two enantiomers between repetitions, in order to minimize systematic errors. The concentration of the peptide in solution was determined using a previously developed fluorometric assay with the fluorescent taggant molecule fluorescamine, a method that is enantiomerically blind.^42^ The results were fit to an apparent first-order mechanism.

### Racemate Kinetics and Enantiomeric Enrichment

A set of reactions with racemic mixtures as reactants was carried out in a Radleys 12-vial parallel reaction system modified with 12 intensity-tuned, voltage-controlled 365 nm LEDs, allowing direct illumination of the vertical plates without interfering with stirring. This system is denoted as “the carousel system”. All vials were simultaneously illuminated following an adsorption equilibrium and were monitored in parallel. Some tests were also performed in the same reactor used for the single-enantiomer kinetics studies.

To quantify the concentration of each enantiomer, a chiral-resolving method was developed for an Agilent 1100 HPLC instrument. An Astec Chirobiotic T (4.6 mm × 15 cm) chiral column, with an isocratic mobile phase of 70% (by volume) methanol and 30% 50 mM triethylamine acetate (TEAA) in water at a pH of approximately 6.75, was used. The flow rate was 0.4 mL/min, and the temperature was set to 20 °C. The run time was 10 min, during which the l enantiomer was the first to elute at 7 min, while the d enantiomer eluted 30 s later. For all measurements the resolution factors were larger than 2.

## Results and Discussion

### Activity Suppression

A functional PED relies on sufficient passivation of the photocatalytic surface around the templating molecules. While thick layers easily accomplish this, it is obvious that too thick a layer is likely to overbridge (encapsulate) the templating molecules, as illustrated in Figure 2C. Hence, it is essential to look for the thinnest layer that sufficiently hampers the photocatalytic activity, while retaining the full spatial information on the templating molecule. As depicted in Figure 3, the expected trend of activity damping with increasing cycle number was observed, resulting in an apparent complete deactivation of the photocatalyst at 12 cycles. The averaged slopes of the graphs in Figure 3 are given in Table S1.

### PED Characterization

PEDs comprised of thick TiO_2_ films on glass, overcoated with Al_2_O_3_, were prepared as described in the Experimental Section and were characterized by XRD, XPS, AFM, and SEM-EDS. The XRD pattern was typical for P25 TiO_2_ (i.e., a mixture of anatase and rutile), as the alumina layers were too thin to have any diffraction effect. The SEM-EDS images (Figure 4) revealed the presence of aluminum atoms at the surface, in quite a homogeneous distribution. As expected, the relative amount of aluminum increased upon increasing the number of ALD growth cycles. It should be noted that all samples, regardless of whether or not they had been overcoated, displayed cracks on their surface (all the way to the glass substrates); however, these cracks are not considered to be a challenge for ALD coating, which is well-suited for irregular, high-aspect-ratio topography.^43^

Table 1 presents the atomic concentration (%), as measured by XPS, of PEDs that had been prepared using imprinting solutions of different concentrations of LeuGly (0, 0.1, 0.5, and 1 mg/mL). In all samples the overcoating layer was prepared by 10 ALD cycles of alumina. All measurements were performed prior to stripping the templating LeuGly. The concentration of oxygen was found to be 56 ± 3%. On a free-carbon basis, the atomic concentration of oxygen was 71%, which is slightly higher than that expected (60–66%), probably reflecting some contribution of oxygenated species adsorbed on the surface. The atomic concentration of carbon was found to be 18 ± 1% for samples prepared with a low concentration of imprinting molecules. The lack of a distinct trend suggests that most of the carbon signal originated from carbonaceous contamination and from ALD-leftover methyl groups rather than from the imprinting molecules. The high atomic concentration of carbon measured in samples prepared with a high concentration of LeuGly suggests that the templating molecules tend to be deposited as aggregates under this condition during preparation. These aggregates may act to ease the further adsorption of organic contaminants. The conclusion regarding the presence of aggregates in samples prepared with the highest concentration of LeuGly is further supported by the low atomic concentration of Ti found in such samples. It should be noted that the XPS measurements were sensitive enough to monitor nitrogen atoms belonging to the LeuGly molecules on the surface; nevertheless, the signal to noise ratio for this rather low concentration of atoms was insufficient for a detailed quantitative analysis, beyond a positive correlation between solution and surface concentrations. For the above reasons, most of the results reported below were obtained with PEDs prepared with a 0.25 mg/mL LeuGly solution.

**Table 1:** XPS Atomic Concentration (%) of Oxygen, Carbon, Titanium, Aluminum and Nitrogen atoms in PEDs Overcoated with 10 ALD Cycles and Templated with 0, 0.1, 0.5, and 1 mg/mL Solutions of l-LeuGly

	atom %			
	0 mg/mL	0.1 mg/mL	0.5 mg/mL	1 mg/mL
O 1s	55.8	57.6	58.3	51.6
C 1s	19.9	18.4	16.7	30.4
Ti 2p3	13.5	14.1	13.8	7.7
Al 2p	10.8	9.8	10.9	9.8
N 1s	∼0	0.2	0.3	0.4

Ultrahigh-resolution AFM measurements were performed on PEDs and on nonimprinted titania films overcoated with alumina (10 ALD cycles), in order to verify the presence of molecular cavities in the PEDs. Unfortunately, the roughness of the surface made it impossible to directly observe such small features (Figure S2). Nevertheless, by a plot of the distribution (ρ) of heights (*z*) relative to the minimum of each frame, a clear difference between the two devices was revealed (Figure 5). Here, the height distribution of the coated, nonimprinted film (denoted (+,−)) peaked at around 2 nm, in a Gaussian manner. In contrast, the height distribution of the imprinted PEDs was considerably wider and could be resolved into two, partially overlapping, distributions: one that was very similar to that of the (+,−) sample and a second that peaked at around a height of 3 nm. The same effect was even more distinct fot the plate prepared with the higher templating molecule concentration, resulting in a bimodal distribution peaking at 2.5 nm and at 4.5 nm. Hence, it may be concluded that the overcoating process did not bridge over the templating molecules, resulting in the formation of molecular cavities on the surface of the PEDs. It should be noted that the data presented in Figure 5 was determined on the basis of 16000 points (50 nm × 50 nm) and hence is definitely statistically significant. Additional distributions measured over smaller areas (Figure S3) show the same trend.

### Single-Enantiomer Kinetics

**Figure 6:**
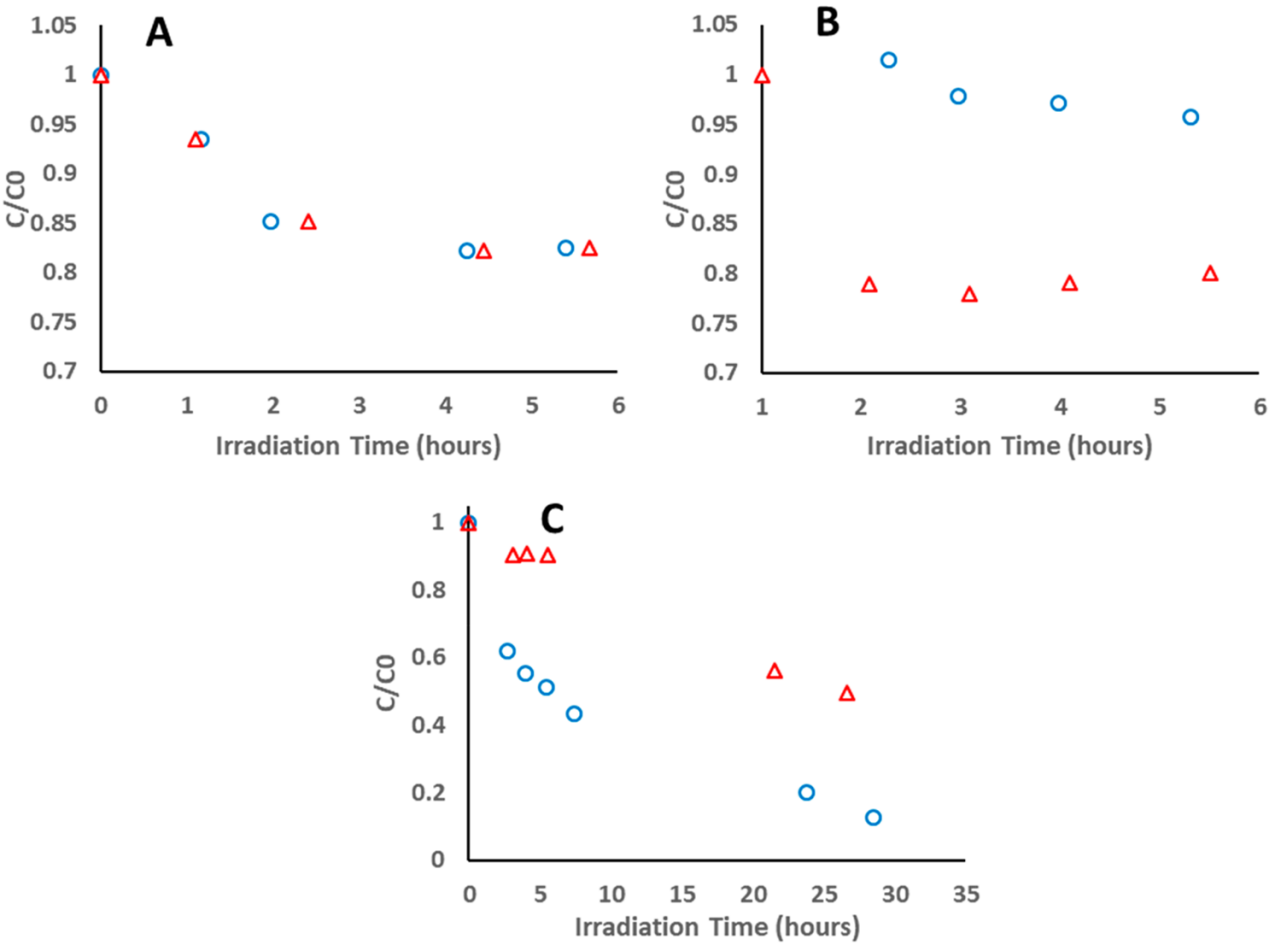
Normalized concentration curves of each single enantiomer test for (A) noncoated, nonimprinted photocatalytic film (−,−), (B) coated, l-imprinted PEDs (+,l), and (C) coated, d-imprinted PEDs (+,d). Red triangles represent the normalized concentration of the l enantiomer, while blue circles represent the normalized concentration of the d enantiomer.

As an initial test of the PEDs, the photocatalytic degradation kinetics of the l enantiomer of LeuGly was compared with that of the d enantiomer in an enantiopure solution. This was done both with PEDs and with nonimprinted, nonovercoated TiO_2_ films. All PEDs reported in this and in the following sections were overcoated with a 10-cycle ALD layer. This thickness was found to yield higher selectivity in comparison to that obtained with imprinted PEDs having thinner layers. As shown in Figure 6A, the degradation kinetics of the l enantiomer were virtually identical with those of the d enantiomer on nonimprinted substrates. In contrast, the degradation kinetics of the l enantiomer were many times faster than those of the d enantiomer on l-imprinted PEDs (Figure 6B). In a symmetrical manner, the degradation kinetics of the d enantiomer were significantly faster than those of the l enantiomer on d-imprinted PEDs (Figure 6C).

To quantify this apparent selectivity effect, the kinetics were fit to a first-order rate law (Table 2). As portrayed in the table, in the absence of imprinted cavities, the rate constant for the degradation of the l enantiomer was almost identical with the rate constant measured for the d enantiomer. This was not the case with the coated, imprinted PEDs. The rate constant of the l enantiomer was significantly higher than that of the d enantiomer upon using l-imprinted PEDs. The opposite was observed upon degrading LeuGly in the presence of d-imprinted PEDs.

**Table 2:** First-Order Rate Constants and l-Enantiomer Selectivities of the Single-Enantiomer Experiment

plate type	l degradation kinetic constant *k*_l_ (1/h)	d degradation kinetic constant *k*_d_ (1/h)
(−,−)	0.0341	0.035
(+,l)	0.0391	0.0107
(+,d)	0.007	0.0624

**Figure 7:**
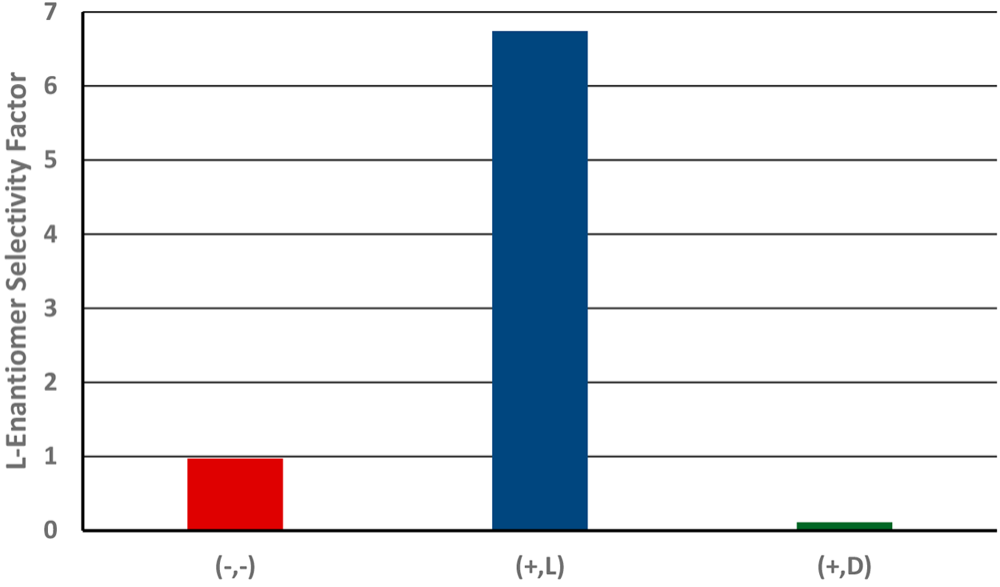
Apparent selectivity factors toward the l enantiomer (*k*_l_/*k*_d_) for the single-enantiomer degradation tests for the noncoated, nonimprinted PED (−,−), the coated, l-imprinted PED (+,l) and the coated, d-imprinted PED (+,d).

The ratios between the rate constants in the degradation of l enantiomer to those of the d enantiomer for the three cases are presented in Figure 7. The differences in the kinetics of the degradation of the d and l enantiomers for the imprinted PEDs are striking. While both types of templates led to preferential degradation of the corresponding enantiomer, the effect of imprinting the d enantiomer was larger than that of imprinting the l enantiomer (9.1 vs 6.7). A second run maintained the enhanced selectivity for the imprinted species, however, with a lower preference, probably due to some incomplete mineralization during the first run.

### Racemate Enrichment

**Figure 8:**
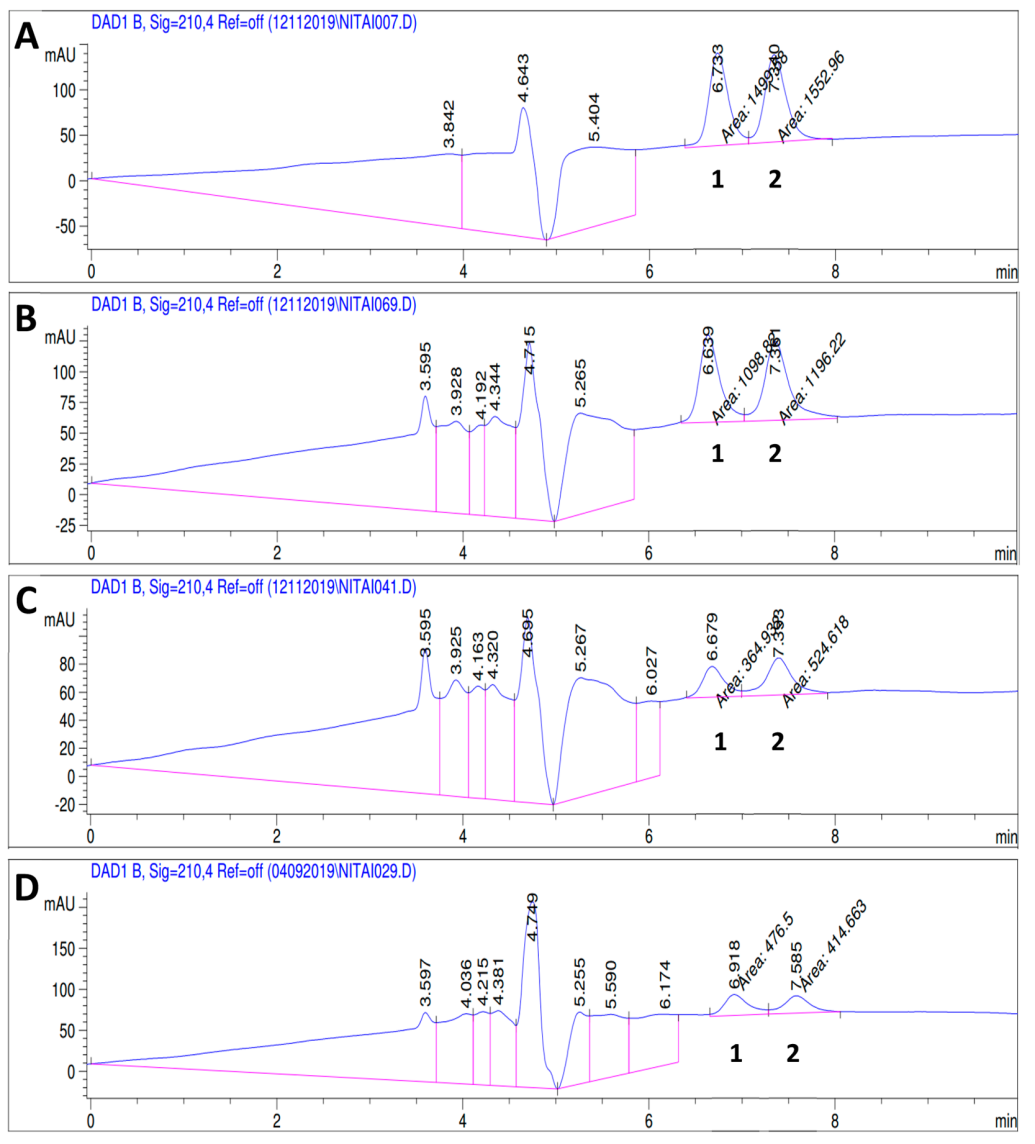
Chromatograms showing the separation of (1) the l enantiomer and (2) the d enantiomer of LeuGly, the degradation products, and the changing ratio between enantiomers upon degradation on the imprinted PEDs: (A) the initial racemic solution; (B) coated, nonimprinted (+,−) PED after 120 h of illumination; (C) coated, l-imprinted (+,l) PED after 95.5 h of illumination; (D) coated, nonimprinted (+,d) PED after 115.5 h of illumination.

After assessing the kinetic differences between the enantiomers in separate reactions, we conducted the ultimate selectivity test—enriching an initially racemic solution by preferential degradation of the templating enantiomer. Figure 8 presents a typical chromatogram of a racemic LeuGly solution, showing the characteristic, well-resolved peaks of the l enantiomer (1) and the d enantiomer (2). As can be seen from the figure, the initially racemic solution (Figure 8A) maintained a constant ratio of the l and d enantiomers and low degradation rates throughout the reaction carried out on the coated, nonimprinted PED (Figure 8B). The reactions carried out on the imprinted PEDs, however, resulted in both a dramatic increase of the degradation rates and an uneven reaction progress, with the templated enantiomer (the distomer) being degraded more quickly than its counterpart (Figure 8C,D).

**Figure 9:**
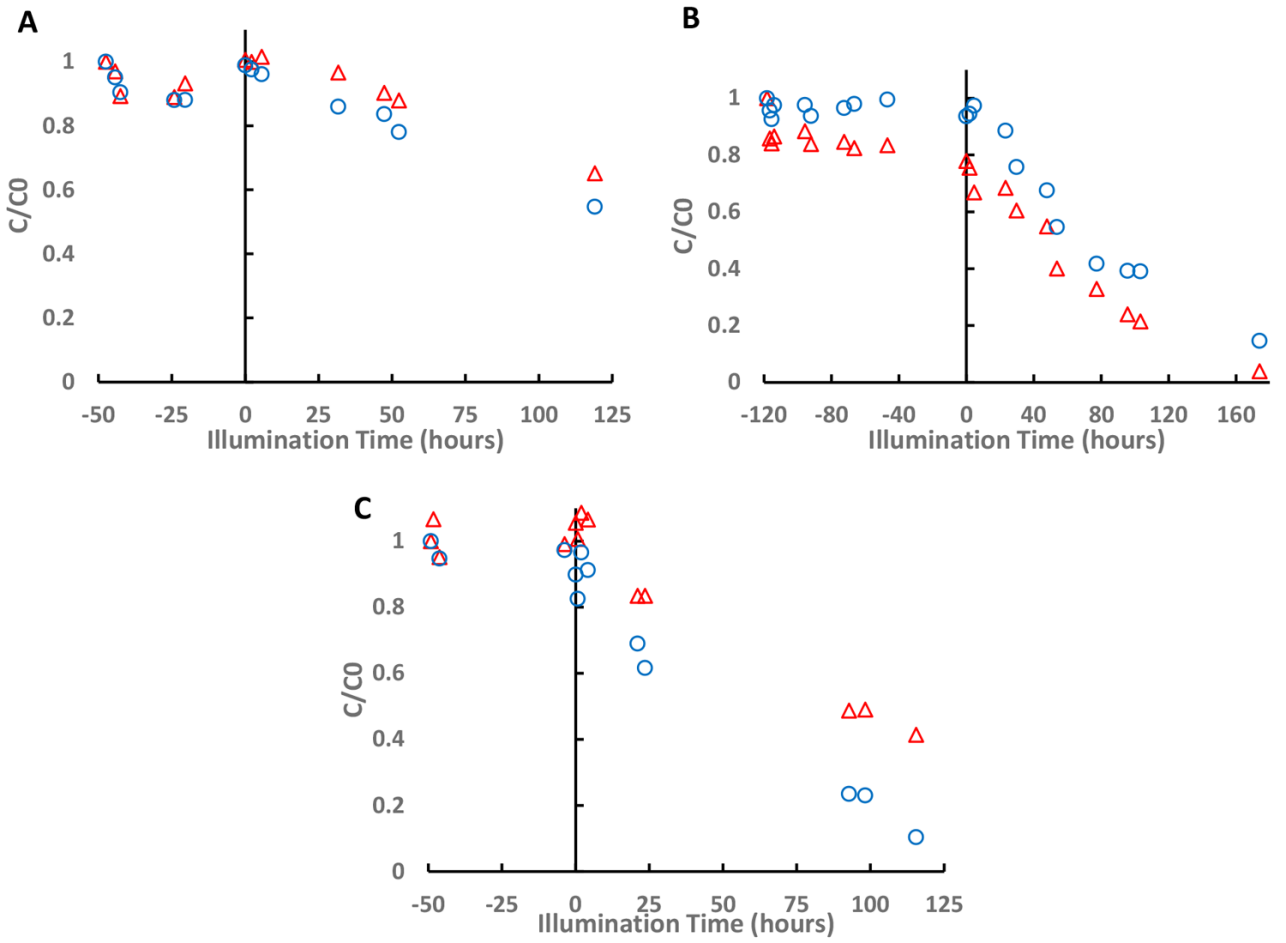
Normalized enantiomeric concentrations during three types of racemate-enrichment tests: (A) nonimprinted, coated PED (+,−); (B) l-imprinted, coated PED (+,l); (C) d-imprinted, coated PED (+,d). Red triangles represent the normalized concentration of the l enantiomer, while blue circles represent the normalized concentration of the d enantiomer. In all traces, negative times represent the “dark” adsorption stage.

Figure 9 shows the concentration of each enantiomer in the racemic mixture, during the dark adsorption stage (negative times) and the subsequent kinetics during illumination of the PEDs. Regardless of the chirality of the templating species, the imprinted PEDs revealed an increased adsorption affinity toward their corresponding distomer (Figure 9B,C), whereas the nonimprinted control device did not exhibit any preference (Figure 9A). This trend also remained during the illumination stage, in which the molecules were photocatalytically degraded. It should be noted that non-negligible degradation of both enantiomers was measured for the coated, nonimprinted devices (Figure 9A). Since the direct photolysis of LeuGly is insignificant on these time scales (as determined in an additional control test), this suggests that the thickness (or conformality) of the alumina overcoating is less than perfect and can be optimized in a manner that may further increase the enrichment performance of the PEDs. Comparing the kinetics of the eutomers (the d enantiomer in the l-imprinted PED and the l enantiomer in the d-imprinted PED) to that of the coated, nonimprinted PED reveals faster degradation kinetics on the imprinted devices. This implies that part of the degradation of the eutomers on the imprinted PEDs occurred within the imprinted cavities. Minimizing such parasitic, residual degradation may involve increasing the thickness of the overcoating layer as well as tuning the conditions (concentration, pH, temperature, etc.) during the templating procedure.

**Figure 10:**
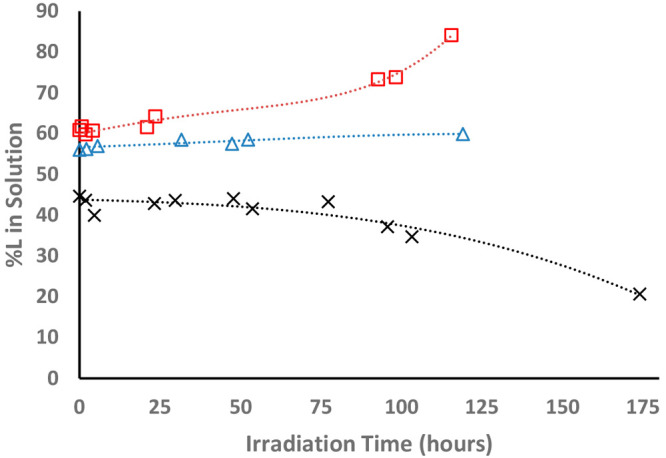
Enrichment curves showing the relative amount of the l enantiomer in each solution during the three types of racemate-enrichment tests: black ×, l-imprinted, coated PEDs; red squares, d-imprinted, coated PEDs; blue triangles, nonimprinted, coated devices.

The preferential adsorption and photocatalytic degradation kinetics are best represented in the form of enrichment curves (Figure 10), displaying the relative concentration of the l enantiomer in the three different types of devices (l imprinted, d imprinted, and nonimprinted) during illumination. Here, an increase in the concentration of the eutomers is clearly observed for both imprinting chiralities. A comparison between d imprinting and l imprinting reveals a quasi-symmetrical behavior, with a slight native tendency toward degradation of the d enantiomer. Sharp-eyed readers may notice that the zero-time ratios between the l and d enantiomers are different from 1. This reflects the preferential selectivity during the “dark” adsorption stage.

**Figure 11:**
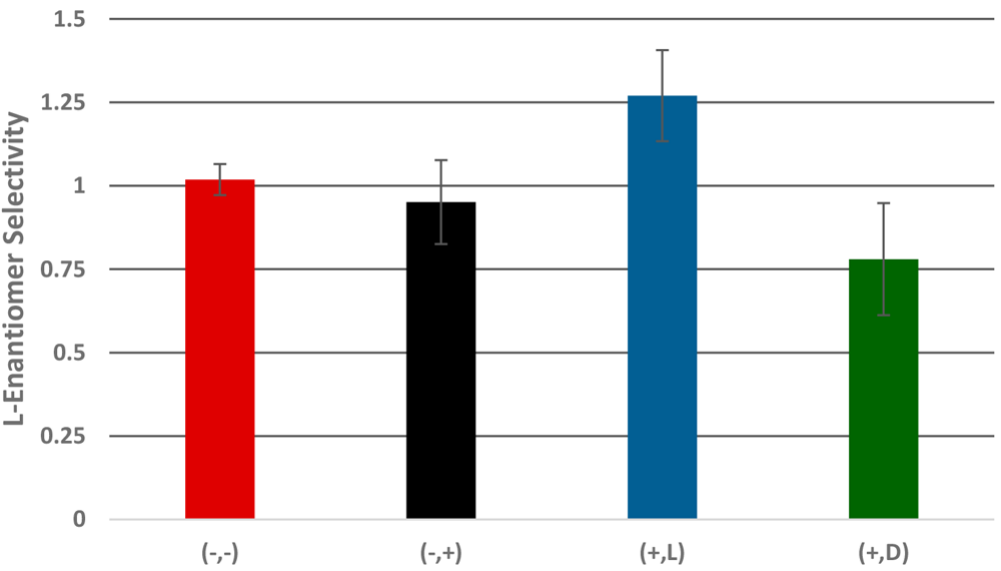
Average values of the l enantiomer selectivity factors (*k*_l_/*k*_d_) for various types of samples, during the racemate-enrichment experiments: (−,−) noncoated, nonimprinted plates; (+,−): coated, nonimprinted plates; (+,l) coated, l-imprinted plates; (+,d) coated, d-imprinted plates.

Figure 11 presents the averaged l enantiomer selectivity factor (i.e., *k*_l_/*k*_d_), obtained upon repeating the set of measurements also in the carousel system described in the Experimental Section, with four types of plates. As previously discussed, this ratio is higher than 1 for the l-imprinted and coated PEDs (1.27 ± 0.1) and lower than 1 for the d-imprinted and coated PEDs (0.78 ± 0.1). As expected, the selectivity factor was very close to unity in the absence of imprinting, for both the coated and noncoated samples.

A comparison between the results of the single-enantiomer experiments (Figure 7) and the results of the racemate-enrichment experiments (Figure 11) shows higher selectivity in the former set of measurements in comparison to that in the latter. Such a tendency was observed also with other photocatalytic systems designed for enhanced selectivity between benzene and 2-methyl-1,4-naphthoquinone.^44^ The reason for the discrepancy could be the presence of a mechanism of adsorption of the eutomer on the distomer (or its incompletely mineralized intermediate product) adsorbed within the cavity, leading to enhanced degradation of the eutomer in the racemate enrichment experiments.

From a practical point of view, successful implementation may require time scales significantly shorter than the typical reaction times reported above. In this context, it should be noted that the use of photocatalytic PEDs in the form of powders is expected to dramatically increase the rates, thus shortening the required reaction time. When a surface area of 50 m^2^ g^–1^ for the powder is taken into account, a conservative calculation gives an estimated rate increase of at least 1 order of magnitude.

## Conclusions

While the holy grail of chiral purification has not yet been found, the device described and tested in this paper shows the promise of a unique new method for the obtainment of enantiopurity. The method avoids the need for expensive and hard to develop methods for asymmetric synthesis or chiral resolution, by utilizing preferential degradation of the distomer molecules within a racemic mixture.

The method, which relies on imprinting the distomer onto the surface of a photocatalyst while passivating the rest of the surface, was shown to induce high selectivity with both enantiopure and racemic mixture solutions. Significant enrichment values were obtained, with values up to 85% of the eutomer in solution (70% enantiomeric excess) at the end of the process, from an initially racemic solution. Nevertheless, the results indicate that the passivating layer did not completely block nonspecific degradation.

The feasibility of using photocatalysis to induce enantiomeric enrichment, demonstrated herein, paves the way for further studies toward optimization and utilization. Two of the crucial factors en route to commercialization are reducing the reaction time and further increasing the selectivity. The number of process parameters governing the selectivity effect is considerable.

Among these parameters are the optimization of the thickness and conformity of the overcoating layer, tuning of the concentration in the templating solution, the use of other photocatalysts (for example, photocatalysts that operate via direct charge transfer), and the introduction of other passivating layers. With respect to reducing the reaction time, it is likely that commercialization may involve the use of particulate PEDs, rather than film-based PEDs that are more convenient for scientific studies. Investigation in these directions is already underway.

## Supporting Information Available

Illustrations of l- and d-LeuGly, ultrahigh-resolution AFM images of the PEDs, AFM height distributions for smaller areas, and kinetic data for the activity reduction measurements of the overcoating layers (PDF)
