# Supplementary material for: Kinetic Resolution of Racemic Mixtures via Enantioselective Photocatalysis
Source: ACS Appl Mater Interfaces. 2021 Aug 11;13(33):39781–90. doi: 10.1021/acsami.1c12216 (PMC8397234; doi:10.1021/acsami.1c12216)
Supplement: Supplementary file 1 — am1c12216_si_001.pdf [file am1c12216_si_001.pdf]

Supporting Information

Kinetic Resolution of Racemic Mixtures via Enantioselective Photocatalysis

Nitai Arbell[a,b], Kesem Bauer[b], Yaron Paz[a,b]*

[a] *The Russell Berrie Nanotechnology Institute, Technion-Israel Institute of Technology, Haifa 3200003, Israel*

[b] *The Wolfson Department of Chemical Engineering, Technion-Israel Institute of Technology, Haifa 3200003, Israel*

**Email: Paz@technion.ac.il*

A B

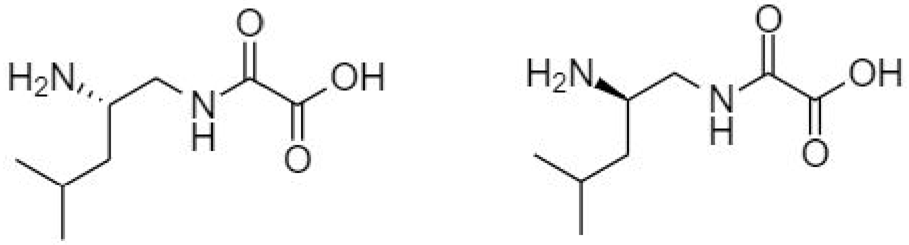

Figure S1. The two enantiomers of LeucylGlycine. (A) shows the line structure of the L-LeuGly enantiomer, while (B)) shows the line structure of the D-LeuGly enantiomer. Illustrations were made using ChemDrawDirect.

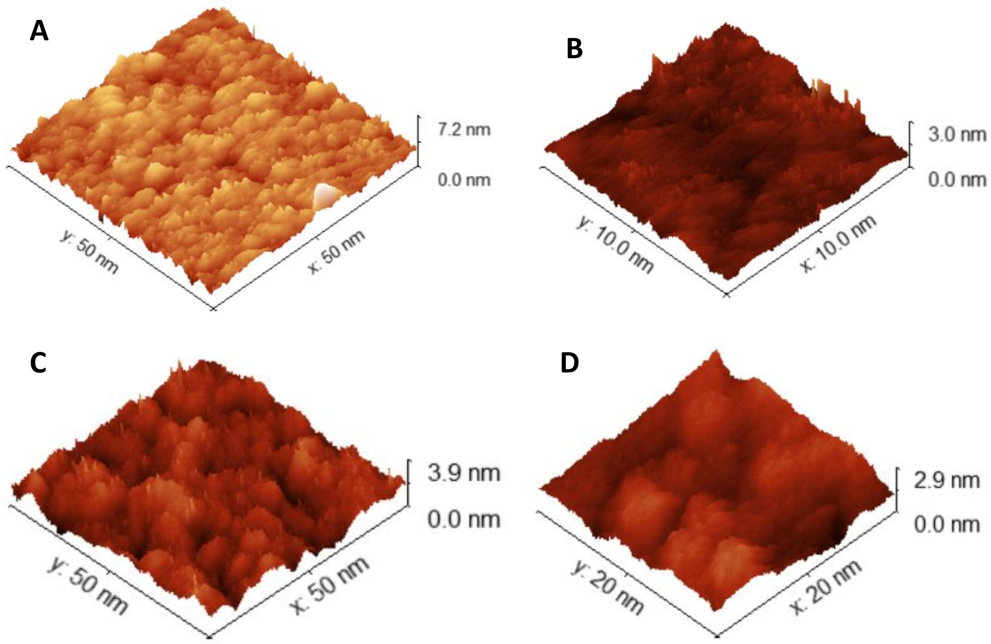

Figure S2. Subnanometric-scale AFM imaging of the PEDs used for the height distribution analysis: (A) coated, non-imprinted (+,-), 50x50 nm area (B) coated, non-imprinted (+,-), 10x10 nm area (C) coated, L-imprinted (+,L), 50x50 nm area (D) coated, L-imprinted (+,L), 20x20 nm area.

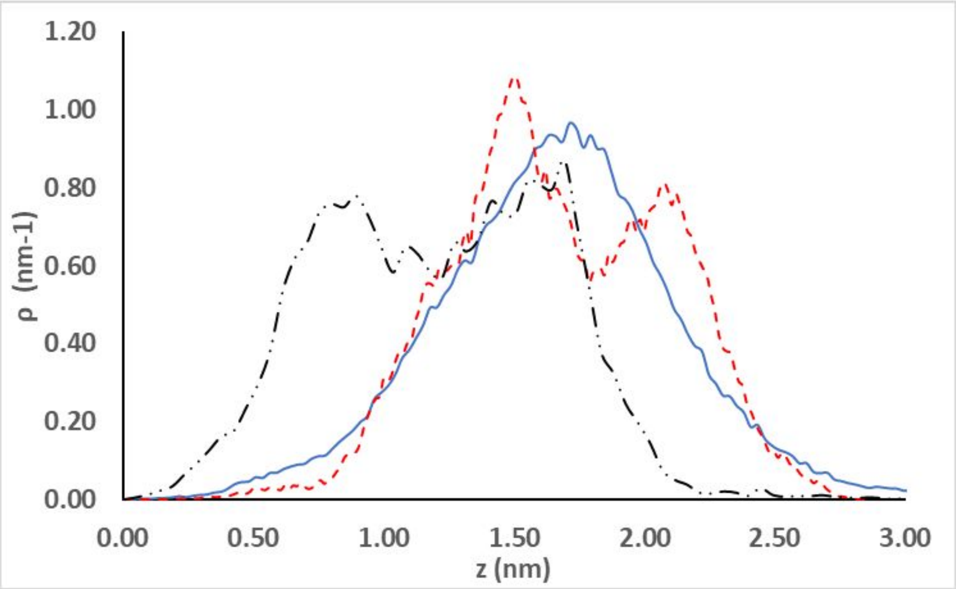

Figure S3. Height distribution functions of the surface of the PEDs as obtained from AFM imaging measured over varying areas. Here, "z" represents the height relative to the minimal height within the frame, while "ρ" is the prevalence. The solid blue line denotes an area of 15x15 nm on the coated, non-imprinted sample (+,-), the dashed red line denotes an area of 20x20 nm on the L-imprinted sample (+,L) templated with an 0.25 mg/ml solution of L-LeuGly, and the dot-dashed black line denotes an area of 10x10 nm on the L-imprinted sample templated with an 0.5 mg/ml solution of L-LeuGly.

Table S1. Average slope of the normalized absobance graphs appearing in Figure 3 of a thin layer of stearic acid deposited on the ALD-coated photocatalytic films as a function of UV-exposure time for different overcoating conditions

Number of ALD cycles and growth temperature	0	4<br>50°C	6<br>50°C	8<br>50°C	8<br>60°C	12<br>50°C
Normalized average slope [1/min]	0.0155	0.0032	0.0025	0.0009	0.0002	0
Activity relative to uncoated TiO_2	100%	20.65%	16.13%	5.81%	1.29%	0
